# Genetic testing among high-risk individuals in families with hereditary nonpolyposis colorectal cancer

**DOI:** 10.1038/sj.bjc.6601529

**Published:** 2004-02-17

**Authors:** M Ponz de Leon, P Benatti, C Di Gregorio, M Pedroni, L Losi, M Genuardi, A Viel, M Fornasarig, E Lucci-Cordisco, M Anti, G Ponti, F Borghi, I Lamberti, L Roncucci

**Affiliations:** 1 Dipartimento di Medicine e Specialità Mediche, Medicina I, Università di Modena e Reggio Emilia, Policlinico, Via del Pozzo 71, Modena 41100, Italy; 2 Divisione di Anatomia Patologica, Ospedale di Carpi, Modena, Italy; 3 Dipartimento di Anatomia Patologica, Università di Modena e Reggio Emilia, Italy; 4 Unità di Genetica Medica, Dipartimento di Fisiopatologia Clinica, Università di Firenze, Italy; 5 Oncologia Sperimentale 1 e Gastroenterologia, Centro di Riferimento Oncologico, Aviano, Pordenone, Italy; 6 Istituto di Genetica Medica, Università Cattolica ‘A.Gemelli’, Roma; 7 Divisione di Gastroenterologia, Ospedale di Viterbo, Italy

**Keywords:** colon, rectum, cancer, tumour, HNPCC, MSI

## Abstract

Hereditary nonpolyposis colorectal cancer (HNPCC) is frequently associated with constitutional mutations in a class of genes involved in DNA mismatch repair. We identified 32 kindreds, with germline mutations in one of three genes hMSH2, hMLH1 or hMSH6. In this study, we purposed to evaluate how many high-risk individuals in each family underwent genetic testing: moreover, we assessed how many mutation-positive unaffected individuals accepted colonoscopic surveillance and the main findings of the recommended follow-up. Families were identified through a population-based registry, or referred from other centres. Members of the families were invited for an education session with two members of the staff. When a kindred was consistent with HNPCC, neoplastic tissues were examined for microsatellite instability (MSI) and immunohistochemical expression of MSH2, MLH1 and MSH6 proteins. Moreover, constitutional mutations were searched by SSCP or direct sequencing of the whole genomic region. Of the 164 subjects assessed by genetic testing, 89 were gene carriers (66 affected – that is, with HNPCC-related cancer diagnosis – and 23 unaffected) and 75 tested negative. Among the 23 unaffected gene carriers, 18 (78.3%) underwent colonoscopy and four declined. On a total of 292 first degree at risk of cancer, 194 (66.4%) did not undergo genetic testing. The main reasons for this were: (a) difficulty to reach family members at risk, (b) lack of collaboration, (c) lack of interest in preventive medicine or ‘fatalistic’ attitude towards cancer occurrence. The number of colorectal lesions detected at endoscopy in gene carriers was significantly (*P*<0.01) higher than in controls (noncarriers). We conclude that a large fraction of high-risk individuals in mutation-positive HNPCC families does not undergo genetic testing, despite the benefits of molecular screening and endoscopic surveillance. This clearly indicates that there are still barriers to genetic testing in HNPCC, and that we are unable to provide adequate protection against cancer development in these families.

Lynch syndrome or hereditary nonpolyposis colorectal cancer (HNPCC) is an inherited disease that accounts for 1–3% of all colorectal malignancies (Lynch and Smyrk, 1996; Peel *et al*, 2000). On a clinical ground, HNPCC is characterised by increased susceptibility to colorectal and several other neoplasms (especially endometrium, ovary, small bowel and urothelium), early age of cancer onset (frequently under 50 years), a proclivity towards tumours in the right colon (from the cecum to the splenic flexure) and frequent multiple primary tumours in the same patient (Lynch and Smyrk, 1996). Molecular investigations have shown that most HNPCC families are associated with constitutional mutations in a class of genes (called hMSH2, hMLH1, hMSH6, hPMS2 and probably others) which are components of a DNA mismatch repair pathway (Wheeler *et al*, 2000; Calvert and Frucht, 2002). As a consequence of their inactivation, cells show a generalised genomic instability, which is particularly evident at microsatellite loci (microsatellite instability, MSI) (Aaltonen *et al*, 1993; Ionov *et al*, 1993; Thibodeau *et al*, 1993).

Since mutations in any of the DNA mismatch repair genes confer a lifetime risk of cancer of 80–85% (Lynch and Smyrk, 1998a,1998b), regular endoscopic controls in individuals at risk (gene carriers) and removal of all premalignant lesions reduce the colorectal cancer rate in HNPCC families (Jarvinen *et al*, 1995). There are objective reasons, therefore, for recommending a close endoscopic surveillance to first-degree relatives of the affected individuals, especially in those subjects in whom this increased susceptibility has been further confirmed by genetic testing.

Despite the potential benefit, genetic testing and the possible identification of gene carriers may represent a source of stress and anxiety in a given family. This is due to the psychological condition of being genetically ‘different’ from the rest of the population, to loss of privacy and possible discrimination in many social activities (Garber *et al*, 1997). Indeed, in a recent investigation, Lerman *et al* (1999) showed that, in four large HNPCC kindreds, more than 50% of the high-risk family members refused further contacts with the investigators or declined genetic counselling. The low rate of acceptance of genetic testing – which was rather unexpected – should raise some concerns on the expected widespread diffusion of this new form of preventive medicine.

Since the availability of genetic testing for hMSH2, hMLH1 and hMSH6 gene mutations, we could identify 32 HNPCC families with germline alterations in one of these genes. In the present investigation, we analysed our study group with three specific objectives: first, to evaluate how many high-risk individuals in each family underwent genetic testing for the search of constitutional mutations; second, to ascertain whether mutation-positive unaffected individuals made a proper use of the test (ie, accepted endoscopic surveillance) and, third, to investigate the main findings of endoscopic surveillance in gene carriers.

## MATERIAL AND METHODS

### Selection of families

Hereditary nonpolyposis colorectal cancer families were assessed in three different centers: the University of Modena (Northern Italy, Region Emilia-Romagna), the Catholic University Medical School in Rome (Central Italy, Region Lazio) and the Aviano Cancer Center (Northern Italy, Region Friuli-Venezia Giulia). In Modena, the families were identified through a multistep approach based on a colorectal cancer registry instituted in 1984 (Ponz de Leon *et al*, 1987; Ponz de Leon *et al*, 1999). All tumours of the large bowel diagnosed in the population (265 227 residents at the 1991 census) were registered. The neoplasms were classified according to the International classification of Diseases for Oncology, 9th revision (ICD-O, 1983). Definitions such as ‘carcinoma *in situ*’, ‘neoplastic foci’ or ‘severe dysplasia’ were not considered as cancer unless there was a clear infiltration of the neoplastic tissue through the muscularis mucosae. Nuclear pedigrees could be obtained in 94% of the registered patients (2462 in the 15-year period 1984–1998). The pedigrees were classified and subdivided according to the presence of clinical criteria indicative of an increased susceptibility to hereditary colorectal cancer (Lynch and Smyrk, 1996). Families showing two or more of these clinical criteria (ie, early age of cancer onset, aggregation of tumours in a sibship, ‘verticality’, proximal location in the large bowel, multiple primaries) were contacted and the pedigrees extended to second- and third-degree relatives. As far as possible, the diagnosis of cancer among family members was verified by histological records, clinical charts or death certificates. In the two other Centres involved in the study (Rome and Aviano), the procedure leading to the identification of HNPCC families was similar to that followed in Modena (ie, definition of nuclear pedigrees, extension of suspected family trees and verification of cancer); the areas, however, were not covered by cancer registries, and individual patients or families with suspicion of HNPCC were referred to the investigators from surgical or endoscopic units operating in those districts. Of the 32 families with constitutional mutations, 25 met the Amsterdam criteria II (Vasen *et al*, 1999) and seven maintained a strong clinical suspicion of HNPCC, because of familial aggregation and early-onset colorectal cancer.

When a kindred showed clinical suspicion of HNPCC, the family doctor was usually contacted for obtaining further information. Subsequently, the proband or other family members were invited by telephone for an education session in one of the centres involved in the study. Families were approached by Internists in Modena, Medical Geneticists in Rome and Gastroenterologists in Aviano. During the session, two members of the staff gathered all relevant information concerning family members (especially cancer development), traced an accurate and extended genealogical tree and required clinical charts or other certifications in order to verify the cancer status. The investigators discussed the general principle of cancer inheritance, advantages and limitations of genetic testing, options of endoscopic surveillance and the possible impact of environmental factors (diet, lifestyle) on cancer-risk reduction. Booklets containing all this information, and further suggestions on diet and style of life, were given to family members at risk. When a kindred was consistent for HNPCC, the successive step was the analysis of microsatellite instability in tumour samples. In the presence of a family history suggestive of Lynch syndrome and of a MSI+ tumour, genetic testing was offered to affected probands from high-risk families, without any charge for the patient and after informed consent. Once a mutation was detected, the family was recontacted and genetic testing was offered to other family members at risk. In most cases, this occurred within 1 year from cancer diagnosis in the proband. Moreover, family doctors, other physicians and collaborative relatives were involved in the attempt to collect further relevant information, particularly with regard to the most distant branches of the family or to individuals living in other regions. Mutation carriers were informed about the need of early colonoscopic surveillance (starting at age 20–25 years), possible benefits and limits of additional screening procedures (endometrial ultrasounds and aspiration biopsy; upper digestive tract endoscopy) and other options aimed at reducing cancer risk (prophylactic interventions, changes in diet and life-style). Noncarriers were reassured, though we made it clear that colorectal cancer is an extremely common disease and that there is large consensus to screen the general population – through fecal occult blood test and lower endoscopy – around the age of 50 years.

### Microsatellite instability

At least one colorectal lesion in each of the 32 families was assessed for MSI, and this was detected in all cases. DNA was extracted from neoplastic and paired normal mucosa, and instability was evaluated with five markers (mono and dinucleotides), as already described (Pedroni *et al*, 1999). Neoplasms showing instability in two or more loci were scored as unstable (MSI+). PCR products from colorectal tumours and the corresponding normal mucosa of the same patient were loaded in adjacent lanes on a standard 6% denaturating polyacrylamide gel, and visualised by auto-radiography.

### MSH2, MLH1 and MSH6 protein expression

Details of the procedure have already been given (Pedroni *et al*, 1999). Briefly, formalin-fixed and paraffin-embedded tumour samples from the affected subjects in each family were sectioned at 6 *μ*m, deparaffined and rehydrated. After antigen retrieval, monoclonal antibodies to full-length hMSH2, hMLH1 and hMSH6 proteins (G168-15, G129-1129, Pharmingen and Clone 44 Transduction Labs, USA) were used at 1 : 40 to 1 : 2000 dilution. Tissue samples were stained using diaminobenzidine as chromogen, according to the Nexes Automatic Staining System (Ventana, Strasburg, France). Lesions were considered positive for protein inactivation when a complete absence of nuclear staining was evident in tumour cells against evident nuclear staining of adjacent normal epithelial and stromal cells.

### Mismatch repair genes mutation analysis

As previously reported (Viel *et al*, 1997), constitutional mutations in the three main DNA mismatch repair genes (hMSH2, hMLH1 and hMSH6) were studied either by single-strand conformation polymorphism (SSCP) or by direct sequencing of the whole genomic region and flanking intron borders using the Big Dye Sequencing Kit (Applera, Foster City, CA, USA) and an applied Biosystem Authomated Sequencer (Applera) on DNA isolated from blood white cells. All families which tested negative by SSCP were subsequently analysed by direct sequencing.

### Statistical analysis

Differences in the occurrence of polyps at endoscopy between gene carriers and noncarriers were evaluated with *Z* tests for independent proportions, when considering the number of patients with or without polyps. When taking into account the number of lesions, their frequency was applied to the appropriate persons/years at risk in the two groups (mutation + and mutation −), and summary *χ*^2^ tests were used – with the Statistical Package for Social Sciences (SPSS) software – to calculate its statistical significance.

## RESULTS

Figure 1

**Figure 1:**
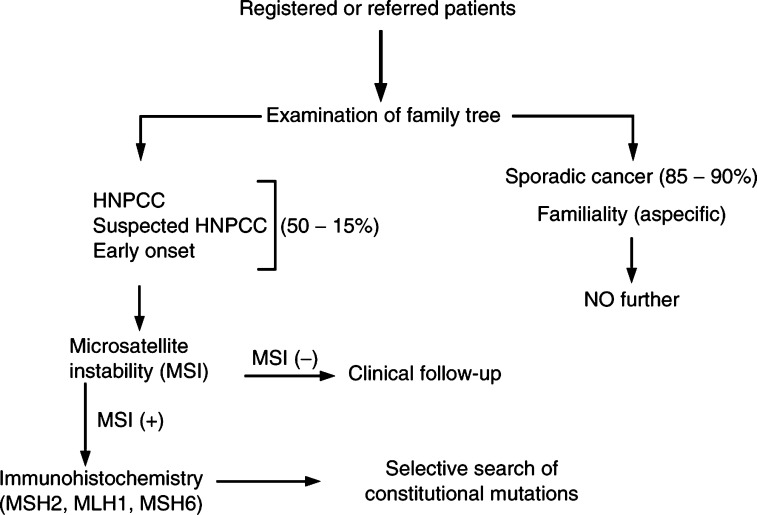
Clinical and molecular strategies developed for the selection and identification of HNPCC. As far as the experience in Modena is concerned (where a specialised colorectal cancer Registry was instituted in 1984), the number corresponding to each category, for the period 1984–1998, are the following. Registered patients: 2462. Sporadic+familial cases: 2207. HNPCC+suspected HNPCC+early-onset cases: 255. MSI+: 53. Lack of protein expression: 21 (immunohistochemistry). Families which underwent genetic testing: 15. Families with constitutional mutations: 12.

shows the strategy that has been followed for the identification of kindreds with inherited colorectal tumours attributable to germline mutations of the main DNA mismatch repair genes. In registered or referred patients, accurate genealogical trees were traced, and, on this basis, a high-risk group for genetic cancer was defined, including HNPCC (according to the Amsterdam Criteria), suspected HNPCC (Park *et al*, 2002) and early-onset (before the age of 50 years) colorectal cancer, representing some 10–15% of all investigated patients with malignancies of the large bowel. As a successive step, microsatellite instability was assessed in this high-risk group; in MSI+ cases, the expression of the protein encoded by the three main mismatch repair genes was evaluated by immunohistochemistry in tumour samples. Lack of protein expression was followed by search of constitutional mutations of the corresponding gene, in the proband and in other family members. With this algorithm, 32 families with germline mutations were identified, between 1994 and 2001.

Details of individual families characterised by constitutional mutations (Viel *et al*, 1997,^1998^; Lucci-Cordisco *et al*, 2001) are shown in Table 1

**Table 1:** Individual families characterised by germline mutations in DNA mismatch repair genes during the period 1994–2001

	**Family**	**Gene**	**Mutation**	**Investigated individuals**	**Mutation positive affected^a^/nonaffected^b^**	**Mutation negative^c^**	**Total individuals at risk^d^**	**Individuals at risk nonevaluated^e^(%)^f^**
1.	MO-1	*hMLH1*	2270ins	10	4/2	4	8	2
2.	MO-2	*hMSH2*	1243–1246del	4	1/1	2	11	8
3.	MO-4	*hMLH1*	IVS17(+5)G>C	4	2/0	2	14	12
4.	MO-10	*hMSH2*	IVS6(+3)A>T	14	5/0	9	32	23
5.	MO-20	*hMLH1*	2270ins	2	2/0	0	12	12
6.	MO-27	*hMLH1*	2270ins	7	4/0	3	12	9
7.	MO-29	*hMLH1*	2270ins	11	2/3	6	21	12
8.	MO-32	*hMSH2*	W345X	1	1/0	0	4	4
9.	MO-39	*hMSH2*	2647del	17	5/1	11	31	19
10.	MO-35	*hMSH2*	IVS5(+3)A>T	6	3/2	1	8	5
11.	MO-41	*hMSH6*	2984del	4	1/1	2	5	2
12.	MO-44	*hMLH1*	1542ins	2	2/0	0	5	5
13.	RM-1	*hMLH1*	Del 2.5 kb	4	3/0	1	5	4
14.	RM-2	*hMLH1*	597–598del	7	2/2	3	11	6
15.	RM-3	*hMSH2*	1497del	3	2/1	0	8	7
16.	RM-4	*hMSH2*	1705insGA	4	1/0	3	3	0
17.	RM-5	*hMLH1*	1520insT	13	4/2	7	15	6
18.	RM-6	*hMLH1*	1846–1848del	4	2/0	2	7	5
19.	AV-24	*hMLH1*	IVS7(-2)A>G	5	2/1	2	11	8
20.	AV-2	*hMLH1*	Q301X	10	2/1	7	8	1
21.	AV-14	*hMLH1*	1783–1784del	3	1/0	2	2	0
22.	AV-20	*hMLH1*	Del 2.5kb	1	1/0	0	2	2
23.	AV-4	*hMSH6*	1960insGTGA	1	1/0	0	0	0
24.	AV-17	*hMSH2*	Q824X	6	2/1	3	6	2
25.	AV-39	*hMSH2*	S473X	4	1/1	2	4	1
26.	AV-28	*hMSH2*	C778X	2	1/0	1	5	4
27.	AV-52	*hMSH2*	399del	7	4/2	1	8	5
28.	AV-56	*hMSH2*	840insT	1	1/0	0	8	8
29.	AV-60	*hMSH2*	IVS6(-2) A>C	1	1/0	0	2	2
30.	AV-68	*hMSH2*	R406X	2	1/1	0	15	14
31.	AV-91	*hMSH2*	Q824X	3	1/1	1	4	2
32.	AV-87	*hMSH2*	C697R	1	1/0	0	5	4
	Total	*hMLH1* (14*)*		164	66/23	75	292	194 (66.4%)
		*hMSH2* (16)						
		*hMSH6* (2)						

a Patients affected by colorectal (or other malignancy of the HNPCC system) at diagnosis.

b First-degree relatives in whom genetic testing showed constitutional mutations.

c First-degree relatives in whom genetic testing showed wild-type gene.

d In each family, first-degree relatives of affected individuals over the age of 20 years.

e In each family, individuals at risk who did not undergo genetic testing.

f Percent of total individuals at risk.

. A total of 164 individuals could be assessed by genetic testing (on average, five per family); 89 were gene carriers (66 affected and 23 nonaffected) and 75 tested negative (ratio: 1.18, not far from the 1 : 1 expected for autosomal dominant transmission). Among the 23 unaffected gene carriers, 19 (82.6%) underwent pancolonoscopy within 1–2 years from the test results, while four declined. Reasons for denial were young age (19 and 21 years, two subjects), ‘lack of time’ associated with some fear of invasive procedures in the other two. On a total of 292 first-degree relatives of affected individuals over the age of 20 years, 194 (66.4%) did not undergo genetic testing. The number of subjects who could be assessed varied from family to family, but was apparently unrelated to the dimension of the kindred, site of origin and level of education. The main reasons for not executing genetic tests were: (a) difficulty (or impossibility) to reach and contact family members (65%); (b) lack of collaboration (15%); (c) lack of interest in preventive (or ‘predictive’) medicine or ‘fatalistic’ attitude towards cancer occurrence or unspecified reasons (20%). Most mutations were found in hMSH2 (16; 50%) or hMLH1 (14; 44%) genes; families MO-1, MO-2, MO-26 and MO-27 showed the same germline alteration. The four families segregating this unusual mutation were resident in the same area, a finding which could be explained by a founder effect (manuscript submitted for publication).

Table 2

**Table 2:** Endoscopic surveillance and tumour occurrence in gene carriers and in controls (mutation negative)

**Groups**	***n***	**Mean age at first endoscopy (years)+s.d.**	**All polyps detected**	**Adenomas**	**No. patients with polyps**	**Other neoplasms developed during follow-up**
Mutation+^a^	19	33.1±8.7	17*	11**	7 (36.8%)***	3 (endometrium, thyroid, colon)
Mutation −b	19	38.5±13	3	3	2 (10.5%)	2 (endometrium, breast)

a Gene carriers, that is, first-degree relatives of affected individuals in whom genetic testing showed the presence of a constitutional mutation.

b Mutation negative, that is, first-degree relatives of affected individuals in whom genetic testing showed wild-type gene.

* *P*<0.001 by summary *χ*^2^ test.

** *P*=0.003 by summary *χ*^2^ test.

*** *Z*=3.22, *P*<0.01.

shows the main endoscopic findings of gene carriers who accepted to be screened. As a control group, we selected those noncarriers who underwent colonoscopy before knowing the test results or despite their low risk status, owing to anxiety or need of more reassuring procedures. Although gene carriers were younger than controls (average difference: 5 years), the number of lesions (adenomatous or hyperplastic polyps, or carcinomas) detected at endoscopy was significantly (*P*<0.001) higher in the carriers. Moreover, colorectal lesions were found in seven high-risk subjects (of 19, 36.8%), but in two controls only (of 19, 10.5%) (*P*<0.01). The length of follow-up was similar in the two groups (4.9±2.6 *vs* 6.5±2.4 years in controls), while the number of colonoscopic investigations/patient was 1.50 in the high-risk group and 1.45 in noncarriers.

## DISCUSSION

The results of the present investigation show that a large fraction of high-risk individuals in mutation-positive HNPCC families does not undergo genetic tests, despite their availability. Among examined subjects, the large majority of gene carriers follows the recommendations, and is willing to undergo colonoscopic surveillance. Despite the relatively young age of gene carriers, the results of endoscopy show an increased risk for polyps and adenomas.

Our findings are similar to those obtained by Lerman and collaborators with American HNPCC families, in whom 43% only of high-risk subjects participated in their counselling and testing program (Lerman *et al*, 1999). Among the reasons for this relative lack of success, the authors gave emphasis to low education and frequent presence of depression symptoms in their study group. It should be noted that the American experience is based on the analysis of four large kindreds, and that most individuals at risk could be contacted directly, by letter or telephone. In our study, 32 families scattered in various Italian regions were analysed, and this prevented us from evaluating the role of strictly personal factors, such as the level of education and presence of depression.

Once the accurate examination of a family tree raises the suspicion of HNPCC, molecular analysis can be undertaken to definitely establish the diagnosis. As recently pointed out by Terdiman (2001), this is not a truly essential step, since the fundamental factor for saving lives is to identify high-risk individuals based on clinical grounds, and to ensure that they receive appropriate surveillance, whether detected to have a mutation or untested. Indeed, the fraction of HNPCC in which mismatch repair gene mutations can be identified ranges between 30 and 60% of all families (the fraction is even lower in the so-called suspected HNPCC), and this means that in approximately half of these kindreds endoscopic surveillance should be initiated simply on a clinical basis (Aaltonen *et al*, 1998; Lynch and Smyrk, 1998a,1998b; Wijnen *et al*, 1998).

However, when a constitutional mutation is found in a HNPCC family, we feel ‘a step forward’, in the sense that high-risk individuals can be precisely identified, and endoscopic surveillance can be limited to gene carriers, thus avoiding unnecessary early investigations in many subjects. It is rather disappointing, therefore, that both the study of Lerman *et al* (1999) and the present investigation clearly show that there are still barriers to the widespread diffusion of genetic testing. In our experience, the main limiting factor was the difficulty in reaching and contacting directly all family members at risk. As indicated by current guidelines, we preferred to adopt a ‘soft’ attitude, avoiding direct telephone calls to distant relatives of the proband and encouraging relatives to talk each other about the risk of cancer running in the family, and the possibilities of prevention and early diagnosis. Moreover, whenever possible, family doctors were contacted and informed about the family history. However, their involvement – though occasionally helpful – had a limited impact in alerting high-risk individuals. It is of interest that the relatively more active attitude of Lerman *et al* (1999) and our less interfering approach gave basically the same results. Lack of collaboration and poor interest in the novel approaches offered by molecular medicine were relevant factors limiting the diffusion of genetic testing also within our HNPCC families. We noticed that a fatalistic attitude to cancer occurrence is still frequent and, surprisingly, often independent by the level of education. To the arguments that cancer was so common among close relatives and that there are tests available for knowing the level of risk, answers such as ‘I do not want to know my risk level, I prefer not to be upset’ or ‘Cancer is so frequent, and there is no way to escape it’, were not uncommon.

The fact that the large majority of mutation carriers underwent endoscopic surveillance is more reassuring. We are not surprised that some individuals hesitated before accepting endoscopy in their 20s or 30s, simply on the basis of a test result. Colonoscopy, even with proper sedation, remains an embarrassing and often painful procedure, which requires a long and disturbing preparation. However, our data suggest that surveillance is acceptable to most, if not all, mutation carriers. Noncarriers can be reassured about their low risk of colorectal cancer and the fact that early colonoscopy is not necessary. Nevertheless, some 30% of them underwent endoscopic controls before the age of 40 years, usually because of anxiety. Since the lifetime risk of colorectal cancer in the Western population is in the order of 5–6% (Black *et al*, 1997), and the same holds true for noncarriers within HNPCC families, we believe that the attention of mutation-negative individuals to the possible event of common malignancies should not be completely discouraged. Indeed, endoscopy in noncarriers showed adenomatous polyps in two of them (10.5%), at an average age of 38 years (Table 2). Finally, the finding of early-onset polyps in one out of three of gene carriers and the frequency of adenomatous lesions are not surprising, and confirm previous investigations (Jarvinen *et al*, 2000; de Vos tot Nederveen Cappel *et al*, 2002). These results further emphasise the importance of a strict endoscopic surveillance in all individuals at risk, in HNPCC families, starting at age 20–25 years (Lindblom and Nordenskjold, 1999, Giardiello *et al*, 2001). Moreover, they reinforce the concept that cancer in these families develops through the adenoma–carcinoma sequence, as in sporadic colorectal cancer.

In conclusion, the main message of the present investigation is that, at present, there are still relevant barriers to genetic testing in HNPCC families and, consequently, we are not able to provide adequate protection against cancer development even in kindreds with identified constitutional mutations. Better education of patients, institution of specialised units entirely devoted to inherited tumours and further collaboration with mass media might help molecular medicine to reach the objective of saving lives (Julian-Reynier *et al*, 1996; Lee *et al*, 2002).

## References

[bib1] Aaltonen LA, Peltomaki P, Leach FS, Sistonen P, Pylkkanen L, Mecklin JP, Jarvinen H, Powell SM, Jen J, Hamilton SR, Petersen GM, Kinzler KW, Vogelstein B, de la Chapelle A (1993) Clues to the pathogenesis of familial colorectal cancer. Science 260: 812–816 8484121 10.1126/science.8484121

[bib2] Aaltonen LA, Salovaara R, Kristo P, Canzian F, Hemminki A, Peltomaki P, Chadwick RB, Kaariainen H, Eskelinen M, Jarvinen H, Mecklin JP, de la Chapelle A (1998) Incidence of hereditary nonpolyposis colorectal cancer and the feasibility of molecular screening for the disease. N Engl J Med 338: 1481–1487 9593786 10.1056/NEJM199805213382101

[bib3] Black RJ, Bray F, Ferlay JM, Parkin DM (1997) Cancer incidence and mortality in the European Union: cancer registry data and estimates of national incidence for 1990. Eur J Cancer 33: 1075–1107 9376190 10.1016/s0959-8049(96)00492-3

[bib4] Calvert PM, Frucht H (2002) The genetics of colorectal cancer. Ann Intern Med 137: 603–612 12353948 10.7326/0003-4819-137-7-200210010-00012

[bib5] de Vos tot Nederveen Cappel WH, Nagengast FM, Griffioen G, Menko FH, Taal BG, Kleibeuker JH, Vasen HF (2002) Surveillance for hereditary nonpolyposis colorectal cancer. A long-term study on 114 families. Dis Colon Rectum 45: 1588–1594 12473880 10.1007/s10350-004-7244-3

[bib6] Garber JE, Offit K, Olopade OI, Fink D, Barbasch A, Barr P, Gleeson RK, Le Stage B (1997) The American Society of Clinical Oncology position on genetic testing. Implications for health care providers – Workshop No. 4. Cancer 80: 632–634 11657063 10.1002/(sici)1097-0142(19970801)80:3+<632::aid-cncr16>3.0.co;2-f

[bib7] Giardiello FM, Brensinger JD, Petersen GM (2001) AGA technical review on hereditary colorectal cancer and genetic testing. Gastroenterology 121: 198–213 11438509 10.1053/gast.2001.25581

[bib8] International Classification of Diseases for Oncology (ICD-O) (1983) In Rilke F (ed), Istocitopatologia WHO 5(suppl.): 1–121

[bib9] Ionov Y, Peinado MA, Malkhosyan S, Shibata D, Perucho M (1993) Ubiquitous somatic mutations in simple repeated sequences reveal a new mechanism for colonic carcinogenesis. Nature 363: 558–561 8505985 10.1038/363558a0

[bib10] Jarvinen HJ, Aarnio M, Mustonen H, Aktan-Collan K, Aaltonen LA, Peltomaki P, de la Chapelle A, Mecklin JP (2000) Controlled 15-year trial on screening for colorectal cancer in families with hereditary nonpolyposis colorectal cancer. Gastroenterology 118: 829–834 10784581 10.1016/s0016-5085(00)70168-5

[bib11] Jarvinen HJ, Mecklin JP, Sistonen P (1995) Screening reduces colorectal cancer rate in families with hereditary nonpolyposis colorectal cancer. Gastroenterology 108: 1405–1411 7729632 10.1016/0016-5085(95)90688-6

[bib12] Julian-Reynier C, Eisinger F, Chabal F, Aurran Y, Nogues C, Vennin P, Bignon YJ, Machelard Roumagnac M, Maugard Louboutin C, Serin D, Versini S, Mercuri M, Sobol H (1996) Cancer genetics clinics: target population and consultees' expectations. Eur J Cancer 32A: 398–403 8814681 10.1016/0959-8049(95)00601-x

[bib13] Lee SC, Bernhardt BA, Helzlsouer KJ (2002) Utilization of BRCA1/2 genetic testing in the clinical setting. Report from a single institution. Cancer 94: 1876–1885 11920551 10.1002/cncr.10420

[bib14] Lerman C, Hughes C, Trock BJ, Myers RE, Main D, Bonney A, Abbaszadegan MR, Harty AE, Franklin BA, Lynch JF, Lynch HT (1999) Genetic testing in families with hereditary nonpolyposis colon cancer. JAMA 17: 1618–1622 10.1001/jama.281.17.1618 10235155

[bib15] Lindblom A, Nordenskjold M (1999) Hereditary cancer. Acta Oncol 38: 439–447 10418710 10.1080/028418699431960

[bib16] Lucci-Cordisco E, Rovella V, Carrara S, Percesepe A, Pedroni M, Bellacosa A, Caluseriu O, Fornasarig M, Anti M, Neri G, Ponz de Leon M, Viel A, Genuardi M (2001) Mutations of the ‘minor’ mismatch repair gene MSH6 in typical and atypical hereditary nonpolyposis colorectal cancer. Familial Cancer 1: 93–99 14574004 10.1023/a:1013872914474

[bib17] Lynch HT, Smyrk T (1996) Hereditary nonpolyposis colorectal cancer (Lynch syndrome). An updated review. Cancer 78: 1149–1167 8826936 10.1002/(SICI)1097-0142(19960915)78:6<1149::AID-CNCR1>3.0.CO;2-5

[bib18] Lynch HT, Smyrk T (1998a) An update of Lynch syndrome (HNPCC). Curr Opin Oncol 10: 349–356 9702403 10.1097/00001622-199807000-00012

[bib19] Lynch HT, Smyrk TC (1998b) Identifying hereditary nonpolyposis colorectal cancer. N Engl J Med 338: 1537–1538 9593794 10.1056/NEJM199805213382109

[bib20] Park JG, Vasen HFA, Park YJ, Park KJ, Peltomaki P, Ponz de Leon M, Rodriguez Bigas MA, Lubinski J, Beck NE, Bisgaard ML, Miyaki M, Wijnen JT, Baba S, Lindblom A, Madlensky L, Lynch HT (2002) Suspected HNPCC and Amsterdam criteria II: evaluation of mutation detection rate, an international collaborative study. Int J Colorectal Dis 17: 109–114 12014418 10.1007/s003840100348

[bib21] Pedroni M, Tamassia MG, Percesepe A, Roncucci L, Benatti P, Lanza Jr G, Gafa R, Di Gregorio C, Fante R, Losi L, Gallinari L, Scorcioni F, Vaccina F, Rossi G, Cesinaro AM, Ponz de Leon M (1999) Microsatellite instability in multiple colorectal cancer. Int J Cancer 81: 1–5 10077143 10.1002/(sici)1097-0215(19990331)81:1<1::aid-ijc1>3.0.co;2-k

[bib22] Peel DJ, Ziogas A, Fox EA, Gildea M, Laham B, Clements E, Kolodner RD, Anton Culver H (2000) Characterization of hereditary nonpolyposis colorectal cancer families from a population-based series of cases. J Natl Cancer Inst 92: 1517–1521 10995807 10.1093/jnci/92.18.1517

[bib23] Ponz de Leon M, Antonioli A, Ascari A, Zanghieri G, Sacchetti C (1987) Incidence and familial occurrence of colorectal cancer and polyps in a health-care district of Northern Italy. Cancer 62: 2848–2859 10.1002/1097-0142(19871201)60:11<2848::aid-cncr2820601141>3.0.co;2-f 3677018

[bib24] Ponz de Leon M, Pedroni M, Benatti P, Percesepe A, Di Gregorio C, Foroni M, Rossi G, Genuardi M, Neri G, Leonardi F, Viel A, Capozzi E, Boiocchi M, Roncucci L (1999) Hereditary colorectal cancer in the general population: from cancer registration to molecular diagnosis. Gut 45: 32–38 10369701 10.1136/gut.45.1.32 PMC1727564

[bib25] Terdiman JP (2001) HNPCC: an uncommon but important diagnosis. Gastroenterology 121: 1005–1008 11606514 10.1053/gast.2001.28634

[bib26] Thibodeau SN, Bren G, Schaid D (1993) Microsatellite instability in cancer of the proximal colon. Science 260: 816–819 8484122 10.1126/science.8484122

[bib27] Vasen HFA, Watson P, Mecklin JP, Lynch HT, the ICG-HNPCC (1999) New clinical criteria for hereditary nonpolyposis colorectal cancer (HNPCC, Lynch syndrome) proposed by the International Collaborative Group on HNPCC. Gastroenterology 116: 1453–1456 10348829 10.1016/s0016-5085(99)70510-x

[bib28] Viel A, Genuardi M, Capozzi E, Leonardi F, Bellacosa A, Paravaton-Petsotas M, Pomponi MG, Fornasarig M, Percesepe A, Roncucci L, Tamassia MG, Benatti P, Ponz de Leon M, Valenti A, Covino M, Anti M, Foletto M, Boiocchi M, Neri G (1997) Characterization of MSH2 and MLH1 mutations in Italian families with hereditary nonpolyposis colorectal cancer. Genes Chromosomes Cancer 18: 8–18 8993976 10.1002/(sici)1098-2264(199701)18:1<8::aid-gcc2>3.0.co;2-7

[bib29] Viel A, Genuardi M, Lucci-Cordisco E, Capozzi E, Rovella V, Fornasarig M, Ponz de Leon M, Anti M, Pedroni M, Bellacosa A, Percesepe A, Covino M, Benatti P, Del Tin L, Roncucci L, Valentini M, Boiocchi M, Neri G (1998) Hereditary nonpolyposis colorectal cancer: an approach to the selection of candidates to genetic testing based on clinical and molecular characteristics. Community Genet 1: 229–236 15178966 10.1159/000016168

[bib30] Wheeler JMD, Bodmer WF, Mortensen NJ (2000) DNA mismatch repair genes and colorectal cancer. Gut 47: 148–153 10861278 10.1136/gut.47.1.148 PMC1727951

[bib31] Wijnen JT, Vasen HFA, Meera Khan P, Zwinderman AH, Van der Klift H, Mulder A, Tops C, Moller P, Fodde R (1998) Clinical findings with implications for genetic testing in families with clustering of colorectal cancer. N Engl J Med 339: 511–518 9709044 10.1056/NEJM199808203390804

